# Department of Surgery

**Published:** 1901-03

**Authors:** W. E. A. Wyman, J. A. Sloan, Robert Jay

**Affiliations:** Milwaukee, Wis.; San Francisco, Cal.; West Liberty, Ia.


### DEPARTMENT OF SURGERY.

By W. E. A. Wyman, M.D.V., V.S.,
MILWAUKEE, WIS.

J. A. Sloan, B.Sc., M.D.V.,
SAN FRANCISCO, CAL.

Robert Jay, M.D.V.,
WEST LIBERTY, IA.

### Hoof Diseases.

(Continued from page 44.)

### *Pododermatitis.*

Pododermatitis Serosa. An acute or chronic, circumscribed or diffuse aseptic inflammation of the pododerm, accompanied with a serous or serofibrinous exudate. The above definition explains itself. Referring to previous discussions, various parts of the pododerm may be involved, as the coronary band, the podophyllous tissue, and the sensitive frog or sole. Diffuse serous pododermatitis is commonly known as founder.

As in most hoof diseases, the causes may be subdivided into predisposing and external ones.

Of the predisposing causes, faulty positions of the limbs and insufficiency of the horny capsule are leading factors. Of the former the base-wide and toe-narrow positions, those camped in front, the low-jointed, sloping fetlock, and the broken-foot axis predispose. In regard to the latter it may be said that hoofs exhibiting insufficiency of hoof, as, for instance, the acutely angled hoof usually does on account of its weak quarters and slightly concaved sole, are also predisposed to pododermatitis. In these the posterior half of the hoof experiences excess of pressure. Furthermore, dry and brittle and contracted hoofs and those with ossified lateral cartilages are predisposed, as in them either wanting elasticity or weakness of the horny box are present ; in fact, the etiology of bruises of the pododerm—that is, corns—will bear repetition in this instance.

The second group—that is, the external or mechanical causes—are of greater importance than the predisposing causes, as the former almost invariably are directly concerned in the creation of the disease. Any external force, therefore, capable of setting up an aseptic pododermatitis is of interest here, viz., concussions, bruises, blows, excessive work on hard or rough ground, long steamer and railroad trips, fast work against the wind, forging, interfering, excessive and unequal paring of the quarters and sole, shoes which are too short or those remaining on too long; finally, heat and cold, while not important factors as direct causes of an aseptic pododermatitis, are, nevertheless, possibilities, as now and then such cases are met with in horseshoeing establishments which one can smell some distance off; in other words, where shoes are fitted too hot.

Eberlein, who handles the subject in a masterly manner, enumerates the following anatomical changes: Serous pododermatitis originates usually in the vascular layer, following in its spread the course of the bloodvessels, thus reaching the phylloid and papillary layer, assisted by the hoof mechanism. The hyperæmic state, with changes in the bloodvessel walls, results in the exudation of a lymph-like, serous or serofibrinous exudate, which, together with more or less cellular infiltration (white and a few red blood-cells), creates an inflammatory œdema of the pododerm, which may be followed by anæmia of the diseased pododerm, simply because this structure lies between two solid, unyielding objects; it cannot expand, its vessels become compressed, and thus the original hyperæmia is replaced by anæmia.

Another influence which is actively concerned in the spread of the inflammation is the sucking and pumping power of the hoof mechanism. By it both exudate and blood-corpuscles are induced to follow the course of the bloodvessels until the surface of the papillary layer is reached. Here the cells of the laminæ imbibe part of the exudate and swell, thus loosening or even separating the sensitive from the horny laminæ.

Acute serous pododermatitis terminates either in resolution, chronic inflammation, purulent inflammation, or necrosis. In cases terminating in resolution the horn cells developed during this period give rise to a horny mass, which is whitish-yellow, translucent, fatty, and glistening. In chronic serous pododermatitis changes involving the pododerm, os pedis, and horny box follow the persistent effects of external and predisposing causes, not to be corrected.

(To be continued.)

---

Dr. Leonard Pearson, State Veterinarian of Pennsylvania, addressed the Farmers' Institute at West Grove, Pa., March 2, 1901.

Dr. J. R. Mehaffy, of Wilmington, Del., met with the loss of his wife and child in the latter part of February.

